# Video-Assisted Thoracoscopic Surgery for Retrieval of an Intrathoracic Knife

**DOI:** 10.1016/j.atssr.2025.06.007

**Published:** 2025-07-09

**Authors:** Angelo Federico, John Hilu, Alexander Restum, Danielle Garcia

**Affiliations:** 1 Department of Thoracic Surgery, Corewell Health, Dearborn, Michigan; 2 Wayne State School of Medicine, Detroit, Michigan

## Abstract

Deliberate foreign body ingestion (DFBI) is a psychopathologic disorder that involves the ingestion of nonnutritive objects to cause self-harm. Depending on the object, the subsequent injuries can be life-threatening if not managed promptly. Whereas the use of video-assisted thoracoscopic surgery (VATS) for the treatment of various lung diseases is well established, the efficacy for retrieval of intrathoracic foreign bodies is unclear and available literature is limited. We report the case of a patient who underwent a VATS for retrieval of an intrathoracic knife after ingestion. This report discusses the use of open thoracotomy vs VATS for intrathoracic foreign body retrieval.

Deliberate foreign body ingestion (DFBI) is a psychopathologic disorder that involves the ingestion of nonnutritive objects to cause self-harm.^1^ The care needed to treat individuals with DFBI is multidisciplinary, with the initial focus being endoscopic or operative removal of the object if it is deemed clinically necessary. There is no “gold standard” for management as the treatment must be individualized on the basis of the location and type of object ingested. The exploration and removal of most intrathoracic foreign bodies revolve around the current standard practice, which is the traditional thoracotomy.^2^ With new advances in thoracic surgery, newer less invasive techniques are becoming increasingly popular.

A 50-year-old woman presented with concern for seizures. The patient had a complex surgical history secondary to a history of DFBI, including multiple esophagogastroduodenoscopies (EGDs) and laparotomies. She endorsed auditory hallucinations that instructed her to swallow foreign objects. This admission was complicated by the ingestion of a plastic knife (Figure 1A). The patient underwent an EGD, in which the knife was visualized within the proximal esophagus below the piriformis fossa. After removal of the knife with grasping forceps, only minor superficial ulcerations of the esophagus were noted. Two days later, a chest radiograph was performed secondary to respiratory distress. This demonstrated another knife and concern for esophageal perforation, given interval development of a right-sided pleural effusion and decreased inflation of the right lung (Figure 1B). Because the patient had acutely increased work of breathing, she was intubated at bedside. Computed tomography scan demonstrated a large right-sided pleural effusion and concern for a radiopaque object partially intraluminal within the esophagus (Figure 2). The patient was then brought emergently to the operating room. EGD revealed a small healed nick at the hypopharynx, suggesting hypopharyngeal penetration and likely tracking of the knife posteriorly into the right side of the chest. A flexible bronchoscopy was then performed and revealed no endobronchial lesions or tears. Given that the foreign body had not been located, the decision was made to perform a right video-assisted thoracoscopic surgery (VATS) for further investigation.

**Figure 1:**
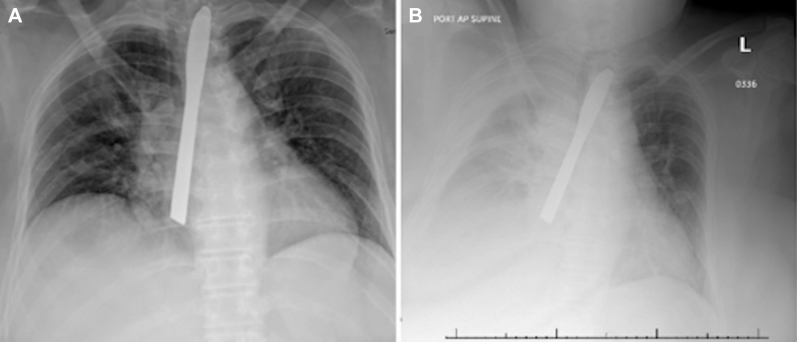
(A) Radiograph demonstrating a foreign body at the level of the esophagus. (B) Radiograph demonstrating a radiopaque knife overlying the midchest, presumed to be within the midesophagus, and interval development of a right-sided pleural effusion.

**Figure 2:**
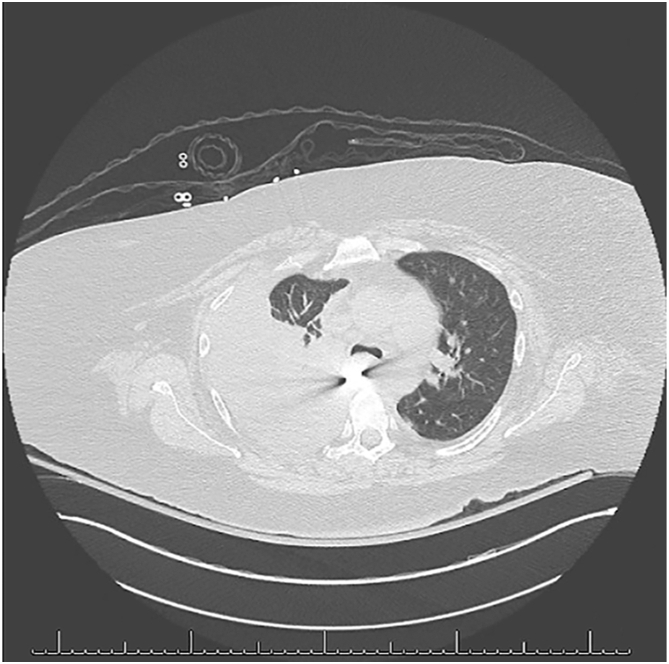
Computed tomography scan showing a large right-sided pleural effusion and concern for a radiopaque object partially intraluminal within the esophagus, extending extraluminally into the pleural space.

The patient was placed in the left lateral decubitus position, and 3 Thoracoports (Medtronic) were placed. As the right lung was decorticated and fibrinopurulent exudate was evacuated, the lung was able to be retracted anteriorly and the knife was visualized (Figure 3A). The knife was tracking under the azygos vein and protruding posteriorly toward the diaphragm, with approximately two-thirds within the pleural space. The serrated edge was under the azygos vein within the extraesophageal tissue with no violation of the esophagus appreciated (Figure 3B). The knife was gently retracted from under the azygos vein and removed through a Thoracoport with a laparoscopic grasper (Figure 3C, 3D). To rule out esophageal perforation or fistula, the nasogastric tube was retracted to the level of the upper esophagus, and methylene blue was injected. After injection, no evidence of dye extravasation was visualized. The procedure was concluded with continued débridement, decortication, and washout of the pleural space. Two chest tubes were then placed within the pleural space, and the right lung expanded appropriately after hemostasis. Direct laryngoscopy was then performed and showed a healing 2-cm vertical laceration to the postcricoid region. The patient progressed well, and an esophagram on postoperative day 11 was unremarkable. The patient was started on an oral diet and discharged to a nursing facility for further psychiatric care.

**Figure 3:**
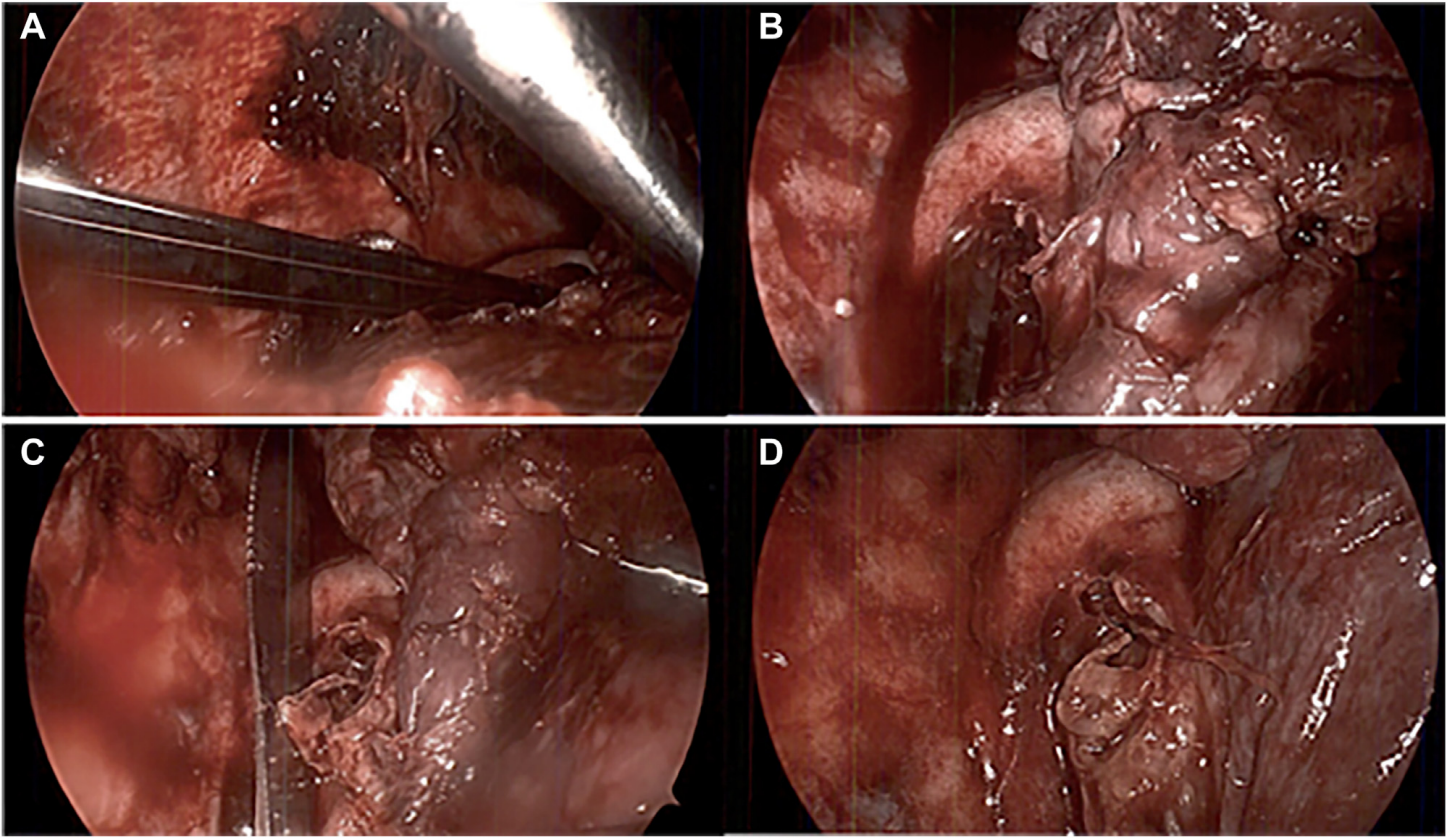
(A) Laparoscopic view of the knife within the pleural space after retraction of the lung. (B) Serrated edge tracking under the azygos vein. (C) Knife after removal from under the azygos vein. (D) View after removal of the knife from the pleural space.

## Comment

Foreign body ingestion is a unique method of self-harm as the ingestion may not be immediately apparent clinically and the chief complaint can vary. The incidence of DFBI has been increasing, specifically in those with psychiatric disorders.^1^ Patients suffering from DFBI are often hospitalized multiple times because of recurrent ingestion; 84% of cases occur in patients with a known prior ingestion, and 85% of these patients have a documented psychiatric history.^3^ Whereas 90% of ingested objects will pass through the gastrointestinal tract, numerous complications can occur.

Endoscopic or surgical removal may be pursued if it is clinically appropriate, especially if the object is long or has sharp edges. Depending on the object, the preferred removal method may vary. The traditional thoracotomy has long been the standard approach for exploration after thoracic trauma as it allows maximal visualization of injuries, control of bleeding, resection of damaged lung tissue, and retrieval of foreign bodies.^4^ As for elective thoracic operations, VATS has been established as the standard approach when it is surgically feasible. The use of VATS has grown during the past 2 decades, changing the surgical approach to many pulmonary, esophageal, and cardiac conditions. VATS has been documented to be superior in terms of incidence of wound and pulmonary complications, postoperative pain, surgical scar satisfaction, shorter hospitalizations, and rate of return to normal lifestyle.^5^ With its increasing popularity, the use has largely focused on certain pathologic processes, such as retained hemothorax and empyema, with less of a focus on foreign body removal.^2^

The available literature comparing traditional thoracotomy vs a thoracoscopic approach for foreign body ingestion is limited. VATS has historically been considered less safe in cases of foreign body ingestion and thus has been underused.^2^ This underuse is likely to be secondary to concern for the meticulous dissection needed to remove objects, especially with sharp edges, and the risk of inadvertent injuries to major pulmonary or cardiac structures. Another potential differentiator is the need for single-lung ventilation. A thoracotomy is possible without 1-lung isolation at the expense of more intraoperative blood loss, chest wall trauma, and potential of undiagnosed injuries or retained foreign bodies due to the hindrance of visualization by the noncollapsed lung.^2^ By use of thoracoscopic evaluation, there is clearer identification of both intrathoracic injuries and retained objects. Although this less invasive technique may offer certain advantages, the overall effectiveness depends on surgeon experience and comfort. Also, a major limitation of VATS is that its use is limited by the hemodynamic instability of a patient. VATS should be restricted to hemodynamically stable patients and those who can tolerate being in a lateral decubitus position.^6^ For hemodynamically unstable patients, there is no clear advantage of VATS over conventional thoracotomy in terms of foreign object removal.^6^

This case demonstrates the use of VATS for successful retrieval of an intrathoracic knife. Whereas the traditional open thoracotomy has long been the standard treatment of intrathoracic foreign body retrieval, this case supports recent research efforts regarding the use of VATS as a safe alternative in hemodynamically stable patients and the potential to minimize intraoperative complications and patient morbidity and to improve overall long-term outcomes.
